# Endothelial activation and stress index predicts 28-day mortality in patients undergoing CRRT

**DOI:** 10.17305/bb.2025.13231

**Published:** 2025-12-08

**Authors:** Jinjin Hu, Junjun Wei, Weiwei Feng, Ling Shi, Chunying Li, Ning Cui, Junxiu Dong, Wei Zhang

**Affiliations:** 1 Hemopurification Center, Ningbo Yinzhou No. 2 Hospital, Ningbo, Zhejiang, China

**Keywords:** Endothelial activation and stress index, EASIX, continuous renal replacement therapy, all-cause mortality, MIMIC-IV

## Abstract

The endothelial activation and stress index (EASIX) is recognized as a prognostic indicator across various diseases; however, its utility in patients undergoing continuous renal replacement therapy (CRRT) is limited. This study aimed to investigate the relationship between EASIX and prognosis in individuals receiving CRRT. Data from patients receiving CRRT were extracted from the Medical Information Mart for Intensive Care IV database. EASIX was calculated and log_2_-transformed. Kaplan–Meier survival analysis was conducted based on log_2_(EASIX) quartiles. Cox proportional hazards regression was utilized to estimate the relationship between EASIX and 28-day all-cause mortality. Potential nonlinear associations were evaluated through restricted cubic splines (RCS) analysis, and subgroup analyses were performed to assess the robustness of EASIX’s impact on all-cause mortality. A total of 2873 intensive care unit patients treated with CRRT were enrolled. Kaplan–Meier analysis revealed that higher EASIX scores were significantly associated with lower 28-day survival (log-rank *P* < 0.001). After adjusting for confounding factors, EASIX remained significantly associated with the risk of 28-day all-cause mortality among CRRT patients (HR: 1.066; 95% CI: 1.026–1.107; *P* ═ 0.001). The area under the curve of the SOFA+EASIX model was 0.694 (95% CI: 0.673–0.714; *P* < 0.001), slightly higher than that of the Sequential Organ Failure Assessment (SOFA) scores alone. These results suggest that EASIX may enhance the predictive performance of SOFA scores. RCS analysis indicated a linear association between log_2_(EASIX) and 28-day all-cause mortality (*P* for overall = 0.001; *P* for nonlinear = 0.224). Subgroup analyses confirmed the robustness of this association across various patient groups. In conclusion, EASIX is independently associated with mortality in patients undergoing CRRT. Prospective studies are warranted to further explore its therapeutic and prognostic significance.

## Introduction

Continuous renal replacement therapy (CRRT) is a critical supportive treatment for patients with acute kidney injury (AKI) [1]. Patients receiving CRRT frequently exhibit severe hemodynamic instability, fluid overload, or complex metabolic disturbances. Epidemiological studies indicate that these patients often possess multiple high-risk factors, such as advanced age, sepsis, and cardiac or pulmonary organ failure [2–4], leading to poor prognoses. A recent cohort study reported a 28-day mortality rate of 62.28% and a 90-day mortality rate of 72.00% among severely ill patients undergoing CRRT [5]. With the increasing recognition of CRRT, its therapeutic scope has expanded to include a wider range of conditions, such as sepsis [1] and multiple organ dysfunction syndrome (MODS) [6]. Consequently, the clinical application of CRRT has been on the rise. A large U.S. cohort study involving 440 hospitals found that among patients hospitalized for sepsis and AKI, an average of 6.4% received renal replacement therapy, with the highest utilization rate reaching 13.4% in a single hospital [7]. This growing utilization of CRRT has inevitably led to increased medical costs and resource consumption. Therefore, effective prognostic assessment of CRRT-treated patients is essential for enhancing clinical outcomes and reducing the economic burden on patients. Independent predictors of survival in CRRT patients include the Charlson comorbidity index, blood urea nitrogen, albumin level, Sequential Organ Failure Assessment (SOFA) score, serum creatinine, mean arterial pressure at CRRT initiation, and phosphate concentration measured 24 h after CRRT initiation [8–10]. Despite the availability of numerous clinical indicators, studies systematically evaluating the impact of endothelial dysfunction on the prognosis of individuals receiving CRRT are still lacking. Endothelial cells, as integral components of the vascular system, function as a barrier between blood and tissue and act as an endocrine organ. They play a central role in maintaining microcirculatory homeostasis by regulating barrier integrity, the balance between coagulation and anticoagulation, vascular tone, inflammatory responses, and immune modulation [11, 12]. Endothelial dysfunction significantly contributes to the development and progression of AKI, sepsis, and other critical illnesses. The endothelial activation and stress index (EASIX) is a recently proposed quantitative tool for assessing endothelial dysfunction. By integrating routine laboratory parameters, such as creatinine, platelet count, and lactate dehydrogenase (LDH), EASIX provides a rapid and convenient reflection of endothelial injury. EASIX has been recognized as a reliable predictor of mortality in various critically ill populations, including those with COVID-19 [13], severe pancreatitis [14], sepsis [15], coronary artery disease [16], atrial fibrillation [17], and heart failure [18]. However, research on EASIX in CRRT populations remains in its early stages, with many aspects yet to be explored. Given that the endothelium represents a “central battlefield” in the pathophysiology of AKI, sepsis, and MODS, endothelial injury may more accurately reflect disease severity than traditional indicators. Moreover, CRRT may influence endothelial function through extracorporeal circulation, highlighting the urgent need for a tailored evaluation system focused on endothelial status.

This research aimed to investigate the relationship between EASIX and 28-day all-cause mortality in CRRT-treated patients using data from the Medical Information Mart for Intensive Care IV database. The findings may have significant implications for enhancing the clinical management of patients undergoing CRRT. Specifically, in resource-limited settings, straightforward indicators may assist in optimizing prognostic assessments and guiding therapeutic decision-making. This study may provide clinicians with a novel tool for early intervention, individualized treatment planning, and improved outcomes, while also offering valuable insights for future research directions.

## Materials and methods

### Study population

The data for this study were obtained from the MIMIC IV database (version 3.1), which includes electronic health records and clinical information for individuals admitted to the intensive care units (ICUs) at Beth Israel Deaconess Medical Center since 2008 [19]. In accordance with MIMIC guidelines, one author (Hu JJ) completed the Collaborative Institutional Training Initiative program (record ID 68836879) and became a credentialed user of PhysioNet. As the information on enrolled patients in this database is de-identified, informed consent was waived. Individuals who received CRRT during their first ICU admission were included in the study. The exclusion criteria comprised: (i) age under 18 years, and (ii) missing baseline measurements of platelet count, creatinine, and LDH.

### Data extraction

Data for this study were extracted from MIMIC-IV (version 3.1) using pgAdmin PostgreSQL tools. Baseline data included patient demographics, vital signs, laboratory parameters, comorbidities, medications and interventions, and assessment scores. The data measured for the first time after ICU admission were selected. Demographic variables encompassed sex, race, age, and body mass index (BMI). Vital signs included respiratory rate (RR), heart rate, diastolic blood pressure (DBP), and systolic blood pressure (SBP). Laboratory data consisted of hemoglobin, red blood cell count (RBC), creatinine, platelet count, white blood cell count (WBC), LDH, aspartate aminotransferase (ALT), albumin, total bilirubin (TBIL), alanine aminotransferase (AST), sodium, potassium, calcium, phosphorus, lactate (Lac), international normalized ratio (INR), oxygen partial pressure (PO2), and prothrombin time (PT). Comorbidities included congestive heart failure (CHF), hypertension, liver disease, and diabetes mellitus (DM). Medications and interventions encompassed statins, norepinephrine, and mechanical ventilation. Assessment scores included the Glasgow Coma Scale (GCS) and the SOFA score.

### EASIX and definition of outcome measures

The EASIX was calculated using the formula: LDH [U/L] × creatinine [mg/dl]/platelet count [10ˆ9/L]. The EASIX calculation process involved several steps: First, patients with incomplete EASIX components were excluded during the screening process. Next, outliers in EASIX components were addressed using the Winsorization method. The 1st percentile (P1) and 99th percentile (P99) of each component were calculated, with values below P1 replaced by P1 and values above P99 replaced by P99. Subsequently, the raw EASIX values were computed according to the formula, and a logarithmic transformation was applied to the raw EASIX values [20]. The primary endpoint of the study was all-cause mortality within 28 days following ICU admission.

### Handling of missing and outlier data

Missing and abnormal data were managed using R (version 4.5.0). Variables with over 20% missing data were excluded (e.g., BMI), while those with less than 20% missingness were imputed through multiple imputation. The random forest method was utilized for imputation, employing 5 imputations and 5 iterations, with the average of the 5 imputation iterations calculated. Outliers were treated through the Winsorization method, wherein values below the 1st percentile were replaced with the 1st percentile value, and values above the 99th percentile were replaced with the 99th percentile value.

### Statistical analysis

The distribution of continuous variables was assessed for normality. Normally distributed data were analyzed using *t*-tests and reported as mean ± standard deviation (SD). Non-normally distributed data were analyzed via the Wilcoxon rank-sum test and presented as median with interquartile range (IQR). Categorical variables were evaluated through the chi-square test and represented by counts and percentages. Individuals were categorized into quartiles based on log_2_(EASIX) (Q1: <1.587, Q2: 1.587–2.727, Q3: 2.727–4.069, Q4: ≥4.069). Kaplan–Meier survival analysis was conducted for these four patient groups, with group differences assessed using the log-rank test.

Cox proportional hazards regression was employed to estimate the relationship between log_2_(EASIX) and 28-day all-cause mortality. The non-adjusted model examined only the univariable effect of log_2_(EASIX) on patient mortality. Model 1 was adjusted for three covariates: race, sex, and age. Model 2 included all covariates from Model 1, with additional adjustments for heart rate, RR, SBP, DBP, albumin, AST, calcium, phosphorus, lactate, sodium, potassium, PO2, PT, RBC, TBIL, WBC, DM, hypertension, liver disease, CHF, statin use, norepinephrine use, mechanical ventilation, GCS, and SOFA score. Additionally, restricted cubic spline (RCS) analysis was conducted to estimate the potential non-linear association between log_2_(EASIX) and survival outcomes.

Subgroup analyses stratified by race, sex, age, DM, liver disease, hypertension, CHF, statin use, norepinephrine use, and mechanical ventilation were performed to evaluate the robustness of the findings. R (version 4.5.0) was employed for the statistical analysis, with a two-sided *P* value of less than 0.05 considered statistically significant.

## Results

### Baseline characteristics

**Figure 1.**
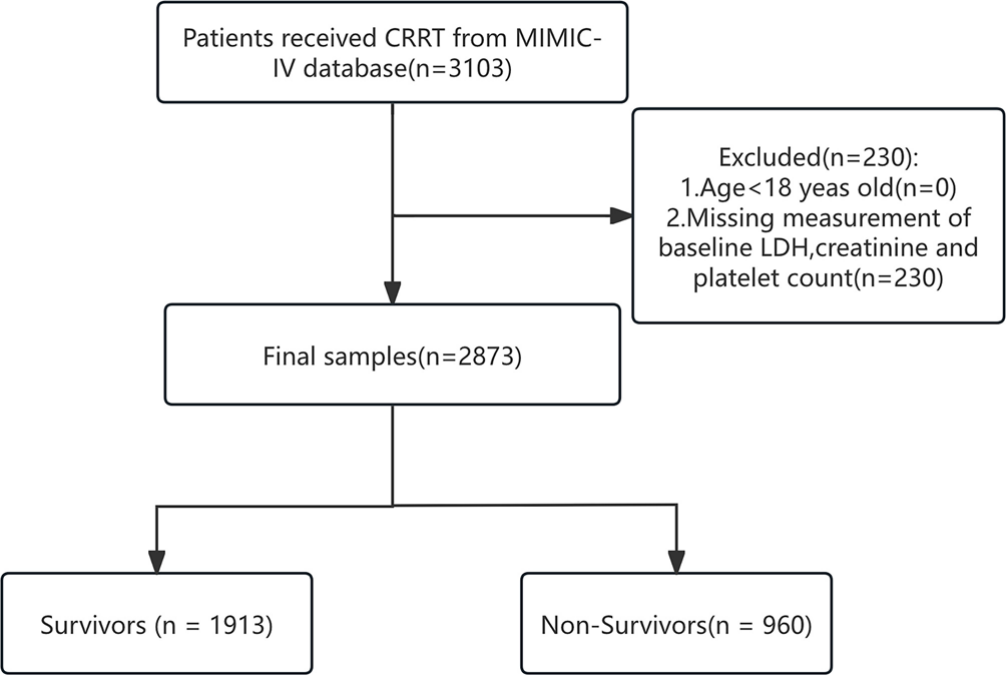
Flowchart illustrating the patient selection process.

A total of 2873 individuals receiving CRRT were enrolled in this study based on the established eligibility criteria (Figure 1). Table 1 presents the baseline characteristics of the study population. The overall 28-day all-cause mortality rate was 33.41%. Among the participants, 58% were over 60 years of age, 61% were male, and 56% were White. During the follow-up period, 1913 patients survived the first 28 days following their initial ICU admission, while 960 patients succumbed to various causes. The missing data rate among survivors (12%) was significantly lower than that among non-survivors (30%) (*P* < 0.001), indicating that the missing racial information was missing not at random (MNAR), potentially linked to the severity of the patients’ conditions.

**Table 1:** Characteristics of the included patients

**Variables**	**Total (*n* ═ 2873)**	**Survivors (*n* ═ 1913)**	**Non-survivors (*n* ═ 960)**	***P***
Age, *n* (%)				<0.001
<60	1199 (42)	850 (44)	349 (36)	
>═60	1674 (58)	1063 (56)	611 (64)	
Gender, *n* (%)				0.7
Male	1750 (61)	1160 (61)	590 (61)	
Female	1123 (39)	753 (39)	370 (39)	
Race, *n* (%)				<0.001
White	1613 (56)	1103 (58)	510 (53)	
Non-White	741 (26)	583 (30)	158 (16)	
Unknown	519 (18)	227 (12)*	292 (30)*	
HR, beats/min, Median (Q1,Q3)	91 (78, 106)	89 (77, 104)	95 (80, 110)	<0.001
RR, beats/min, Median (Q1,Q3)	20 (16, 24)	19 (16, 24)	21 (17, 26)	<0.001
SBP, mmHg, Median (Q1,Q3)	113 (97, 130)	115 (100, 131)	109 (93, 128)	<0.001
DBP, mmHg, Median (Q1,Q3)	55 (47, 65)	56 (48, 65)	54 (45, 64)	<0.001
WBC, K/uL, Median (Q1,Q3)	11.9 (7.8, 17.3)	11.2 (7.5, 15.9)	13.5 (8.9, 20.1)	<0.001
RBC, m/uL, Median (Q1,Q3)	3.21 (2.7, 3.83)	3.21 (2.72, 3.8)	3.21 (2.67, 3.89)	0.894
Hemoglobin, g/dL, Median (Q1,Q3)	9.7 (8.2, 11.4)	9.6 (8.2, 11.3)	9.7 (8.07, 11.53)	0.552
Platelet, K/uL, Median (Q1,Q3)	160 (100, 236)	165 (106, 238)	148 (86, 231.75)	<0.001
Creatinine, mg/dL, Median (Q1,Q3)	2.3 (1.3, 4.4)	2.3 (1.2, 4.7)	2.3 (1.4, 3.9)	0.55
LDH, IU/L, Median (Q1,Q3)	359 (247, 656)	320 (230, 533)	490.5 (308, 959.5)	<0.001
Albumin, g/dL, Median (Q1,Q3)	3 (2.5, 3.4)	3 (2.6, 3.5)	2.8 (2.3, 3.3)	<0.001
Tbil, mg/dL, Median (Q1,Q3)	0.8 (0.4, 2.6)	0.7 (0.4, 2)	1.2 (0.6, 4.5)	<0.001
ALT, IU/L, Median (Q1,Q3)	33 (17, 97)	28 (16, 70)	47 (23, 151.25)	<0.001
AST, IU/L, Median (Q1,Q3)	59 (29, 186)	48 (25, 129)	101 (42.75, 330.5)	<0.001
Sodium, mEq/L, Median (Q1,Q3)	137 (134, 141)	138 (134, 140)	137 (133, 141)	0.566
Potassium, mmol/L, Median (Q1,Q3)	4.4 (3.8, 5.1)	4.3 (3.8, 5.1)	4.4 (3.8, 5.03)	0.792
Calcium, mg/dL, Median (Q1,Q3)	8.3 (7.7, 8.9)	8.3 (7.7, 8.9)	8.2 (7.6, 8.8)	0.024
Phosphorus, mmol/L, Median (Q1,Q3)	4.6 (3.5, 6.2)	4.4 (3.4, 5.8)	5.1 (3.7, 7)	<0.001
Lac, mmol/L, Median (Q1,Q3)	2 (1.3, 3.7)	1.8 (1.2, 3)	2.8 (1.7, 5.5)	<0.001
PO2, mmHg, Median (Q1,Q3)	87 (52, 170)	91 (53, 185)	80 (51, 146.25)	<0.001
INR, Median (Q1,Q3)	1.5 (1.2, 1.9)	1.4 (1.2, 1.8)	1.6 (1.3, 2.3)	<0.001
PT, sec, Median (Q1,Q3)	16 (13.4, 21.1)	15.3 (13.1, 19.5)	17.9 (14.1, 25.52)	<0.001
Diabetes, *n* (%)				<0.001
No	1891 (66)	1175 (61)	716 (75)	
Yes	982 (34)	738 (39)	244 (25)	
Hypertension, *n* (%)				<0.001
No	1599 (56)	978 (51)	621 (65)	
Yes	1274 (44)	935 (49)	339 (35)	
Liver disease, *n* (%)				0.284
No	1735 (60)	1169 (61)	566 (59)	
Yes	1138 (40)	744 (39)	394 (41)	
Congestive heart failure, *n* (%)				<0.001
No	1334 (46)	774 (40)	560 (58)	
Yes	1539 (54)	1139 (60)	400 (42)	
Statins, *n* (%)				<0.001
No	1019 (35)	490 (26)	529 (55)	
Yes	1854 (65)	1423 (74)	431 (45)	
Norepinephrine, *n* (%)				<0.001
No	806 (28)	589 (31)	217 (23)	
Yes	2067 (72)	1324 (69)	743 (77)	
Mechanical ventilation, *n* (%)				0.472
No	655 (23)	428 (22)	227 (24)	
Yes	2218 (77)	1485 (78)	733 (76)	
GCS, Median (Q1,Q3,%**)	15 (15, 15, 16.92)	15 (15, 15, 15.37)	15 (15, 15, 20.00)	0.011
SOFA, Median (Q1,Q3)	9 (6, 12)	8 (5, 11)	11 (8, 14)	<0.001
Survival time in 28d, Median (Q1,Q3)	28 (16, 28)	28 (28, 28)	8 (4, 16)	<0.001
EASIX, Median (Q1,Q3)	6.62 (3, 16.79)	5.62 (2.62, 13.55)	9.02 (4.21, 26.31)	<0.001
log_2_(EASIX), Median (Q1,Q3)	2.73 (1.59, 4.07)	2.49 (1.39, 3.76)	3.17 (2.08, 4.72)	<0.001
Quartile of log_2_(EASIX), *n* (%)				<0.001
Q1	718 (25)	560 (29)	158 (16)	
Q2	718 (25)	497 (26)	221 (23)	
Q3	718 (25)	463 (24)	255 (27)	
Q4	719 (25)	393 (21)	326 (34)	

Data are presented as the mean ± standard deviation (SD) for normally distributed variables, median (interquartile range, IQR) for skewed variables, and counts (proportions) for categorical variables. The quartiles are defined as follows: Q1 (<1.587), Q2 (1.587–2.727), Q3 (2.727–4.069), and Q4 (≥4.069). *Note: The “missing race information rate for each group” refers to the proportion of the “Unknown” category within the total population. The missing rate among survivors (12%) was significantly lower than that among non-survivors (30%), *P* < 0.001. **Note: The percentage indicates the proportion of GCS scores < 14. Abbreviations: EASIX: Endothelial activation and stress index; HR: Heart rate; RR: Respiratory rate; SBP: Systolic blood pressure; DBP: Diastolic blood pressure; WBC: White blood cell; RBC: Red blood cell; LDH: Lactate dehydrogenase; TBIL: Total bilirubin; ALT: Alanine aminotransferase; AST: Aspartate aminotransferase; PT: Prothrombin time; GCS: Glasgow Coma Scale; SOFA: Sequential Organ Failure Assessment; INR: International normalized ratio.

Comparisons between survivors and non-survivors revealed significant differences in race, vital signs, age, SOFA score, and GCS score. Survivors were generally younger, exhibited lower heart rates and RRs, and had higher systolic and DBP compared to non-survivors (all *P* < 0.001). Furthermore, the prevalence of DM, hypertension, and CHF was higher among survivors than non-survivors. In terms of laboratory parameters, both EASIX and log_2_(EASIX) values were significantly lower in survivors compared to non-survivors. Survivors also demonstrated higher platelet counts, albumin levels, calcium levels, and PO2, while having lower WBC, LDH, TBIL, ALT, AST, phosphorus, lactate, INR, and PT levels. In the survivor group, the use of statins was more prevalent, while norepinephrine administration was less frequent, with no significant difference in mechanical ventilation use.

### Kaplan–Meier survival curve

**Figure 2.**
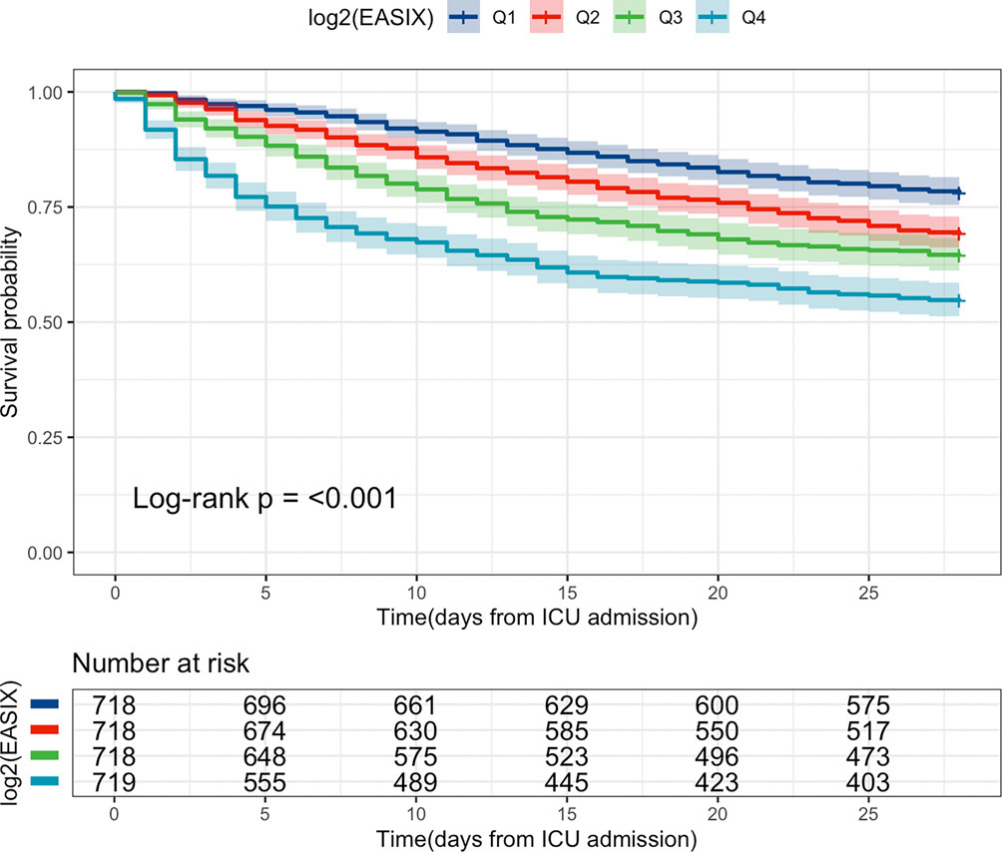
**Kaplan–Meier curves for 28-day survival stratified by quartiles of log_2_(EASIX).** Patients in higher EASIX quartiles (Q2–Q4) showed progressively lower 28-day survival compared with the lowest quartile (Q1), with the greatest all-cause mortality observed in Q4 (log-rank *P* < 0.001). Numbers at risk for each quartile are shown below the *x*-axis. Abbreviation: EASIX: Endothelial activation and stress index.

Survival analysis was conducted based on the quartiles of log_2_(EASIX) (Figure 2). The analysis indicated that, compared to Q1, individuals in the higher EASIX quartiles (Q2, Q3, and Q4) exhibited significantly lower 28-day survival rates (log-rank *P* < 0.001), with the highest all-cause mortality observed in the top EASIX quartile (Figure 2).

### Relationship between EASIX and 28-day all-cause mortality among CRRT-treated patients

Several Cox regression models were developed to assess the independent effect of EASIX on 28-day all-cause mortality among patients undergoing CRRT. Prior to conducting the Cox regression analysis, we performed a multicollinearity diagnostic assessment on all variables (Supplementary Material 1). Preliminary analysis revealed a correlation between AST and ALT, with variance inflation factor (VIF) values of 5.28 and 4.93, respectively, indicating the presence of multicollinearity. To mitigate this issue, we retained the AST variable and excluded ALT from the final model, resulting in VIF values for all remaining variables dropping below 5. Furthermore, the model was validated through the Schoenfeld residual test to confirm the validity of the proportional hazards assumption, thereby reinforcing the reliability of the continuous variable estimates (Supplementary Material 2 and 3).

**Table 2:** Risk of 28-day mortality based on log_2_-EASIX

	**Non-adjusted**		**Model 1**		**Model 2**		**Model 3**	
	**HR (95% CI)**	***P* value**	**HR (95% CI)**	***P* value**	**HR (95% CI)**	***P* value**	**HR (95% CI)**	***P* value**
log_2_(EASIX)	1.191 (1.156–1.226)	<0.001	1.190 (1.155–1.226)	<0.001	1.066 (1.026–1.107)	0.001	1.121 (1.081–1.162)	<0.001
*Quartile of log_2_(EASIX)*								
<1.587	Reference		Reference		Reference		Reference	
1.587∼2.727	1.474 (1.202–1.808)	<0.001	1.407 (1.147–1.727)	0.001	1.184 (0.961–1.459)	0.110	1.301 (1.057–1.602)	0.013
2.727∼4.069	1.821 (1.493–2.221)	<0.001	1.761 (1.443–2.148)	<0.001	1.273 (1.032–1.571)	0.024	1.456 (1.182–1.793)	<0.001
≥4.069	2.666 (2.204–3.224)	<0.001	2.565 (2.193–3.218)	<0.001	1.478 (1.185–1.844)	<0.001	1.896 (1.530–2.349)	<0.001
*P* for trend		<0.001		<0.001		<0.001		<0.001

Model 1: adjusted for age, gender, and race. Model 2: adjusted for Model 1 plus HR, RR, SBP, DBP, albumin, AST, calcium, phosphorus, Lac, sodium, potassium, PO_2_, PT, RBC, TBIL, WBC, diabetes, hypertension, liver disease, CHF, statins, norepinephrine, mechanical ventilation, GCS, and SOFA. Model 3: adjusted as in Model 2, but with SOFA removed. Continuous HRs are reported per 1-unit increase in log_2_(EASIX). Abbreviations: HR: Heart rate; RR: Respiratory rate; SBP: Systolic blood pressure; DBP: Diastolic blood pressure; Lac: Lactate; PO_2_: Partial pressure of oxygen; PT: Prothrombin time; RBC: Red blood cell; TBIL: Total bilirubin; WBC: White blood cell; CHF: Congestive heart failure; GCS: Glasgow Coma Scale; SOFA: Sequential Organ Failure Assessment; EASIX: Endothelial activation and stress index.

**Figure 3.**
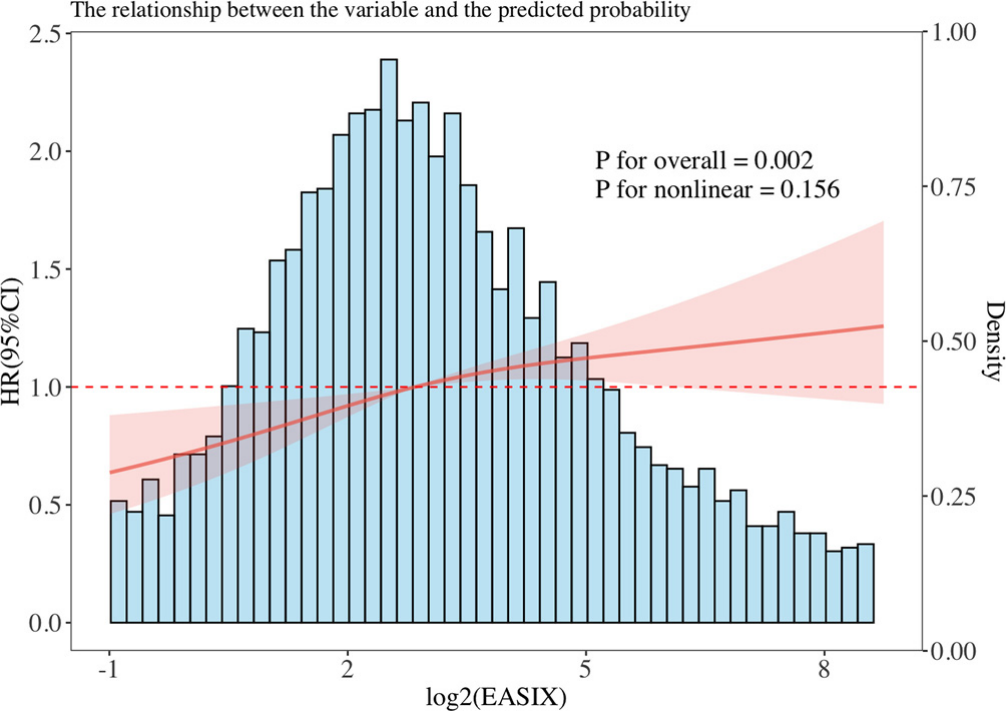
**RCS analysis of the association between log_2_(EASIX) and 28-day all-cause mortality in CRRT-treated ICU patients.** The solid red line depicts the adjusted hazard ratio, with the shaded area indicating the 95% confidence interval; the horizontal dashed line represents the reference value (HR = 1.0). The histogram illustrates the distribution of log_2_(EASIX) in the study cohort. RCS analysis showed a significant overall association between log_2_(EASIX) and 28-day all-cause mortality (*P* for overall = 0.001) with no evidence of nonlinearity (*P* for nonlinearity = 0.224). Abbreviations: RCS: Restricted cubic spline; EASIX: Endothelial activation and stress index; CRRT: Continuous renal replacement therapy; ICU: Intensive care unit; HR: Hazard ratio; *P*: *P* value.

As shown in Table 2, in the unadjusted model, the continuous variable log_2_(EASIX) was positively associated with 28-day all-cause mortality (hazard ratio (HR): 1.191, 95% CI: 1.156–1.226, *P* < 0.001). The multivariable Cox regression analysis indicated that, after adjusting for confounders, each 1-unit increase in log_2_(EASIX) correlated with a 19% increase in all-cause mortality in Model 1 (HR: 1.190, 95% CI: 1.155–1.226) and a 6.6% increase in Model 2 (HR: 1.066, 95% CI: 1.026–1.107). When stratified into quartiles based on log_2_(EASIX) (Q1: <1.587, Q2: 1.587–2.727, Q3: 2.727–4.069, Q4: ≥4.069), the remaining three quartiles exhibited significantly higher 28-day all-cause mortality compared to the lowest quartile (Q1 as reference, *P* for trend < 0.001): Q2 (HR: 1.474, 95% CI: 1.202–1.808), Q3 (HR: 1.821, 95% CI: 1.493–2.221), and Q4 (HR: 2.666, 95% CI: 2.204–3.224). After adjustment in Model 2, the change in all-cause mortality risk in Q2 compared to adjusted Q1 was not statistically significant (HR: 1.184, 95% CI: 0.961–1.459, *P* ═ 0.11), whereas Q3 (HR: 1.273, 95% CI: 1.032–1.571, *P* ═ 0.024) and Q4 (HR: 1.478, 95% CI: 1.185–1.844, *P* < 0.001) continued to demonstrate significantly increased mortality risk (Q1 as reference, *P* for trend < 0.001). Lastly, since the SOFA score incorporates platelet count and kidney function—components of the EASIX calculation—it may influence the efficacy of EASIX through over-regulation of mediating variables or collinearity. Consequently, we developed Model 3, which excluded the SOFA covariate compared to Model 2. The results indicated that in Model 3, each unit increase in log_2_(EASIX) was associated with a 12.1% increase in mortality risk (HR: 1.121, 95% CI: 1.081–1.162, *P* < 0.001). Moreover, Q2 (HR: 1.301, 95% CI: 1.057–1.602, *P* ═ 0.013), Q3 (HR: 1.456, 95% CI: 1.182–1.793, *P* < 0.001), and Q4 (HR: 1.896, 95% CI: 1.530–2.349, *P* < 0.001) exhibited higher mortality risks compared to Q1, with statistically significant differences. Furthermore, the dose-response relationship between EASIX and the risk of 28-day all-cause mortality was assessed using RCS. RCS analysis demonstrated a linear association between log_2_(EASIX) and 28-day all-cause mortality (*P* for overall = 0.001; *P* for nonlinear = 0.224) (Figure 3).

### Subgroup analysis

Subgroup analyses were conducted based on race, sex, age, DM, hypertension, liver disease, CHF, statin use, norepinephrine use, and mechanical ventilation to further evaluate the robustness of the relationship between EASIX and 28-day all-cause mortality.

**Figure 4.**
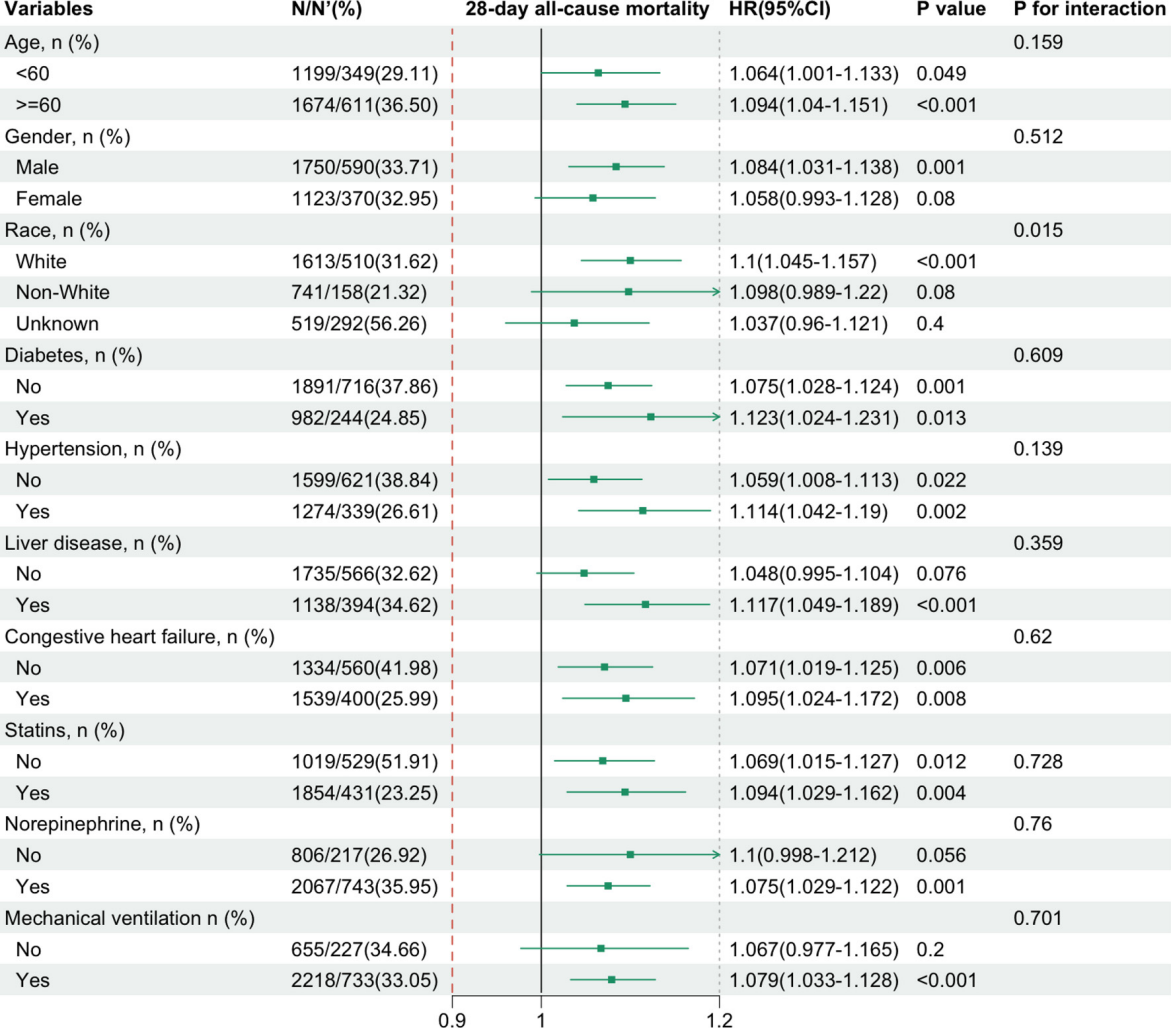
**Subgroup analyses of the association between log_2_(EASIX) and 28-day all-cause mortality in CRRT-treated patients**. The forest plot shows adjusted hazard ratios with 95% confidence intervals per 1-unit increase in log_2_(EASIX) across subgroups defined by age, gender, race, diabetes, hypertension, liver disease, CHF, statin use, norepinephrine use, and mechanical ventilation, with corresponding *P* values and *P* for interaction. Abbreviations: EASIX: Endothelial activation and stress index; CRRT: Continuous renal replacement therapy; CHF: Congestive heart failure.

Both subgroup and interaction analyses confirmed that the positive correlation between EASIX and 28-day all-cause mortality in CRRT-treated individuals was consistent across most subgroups (*P* for interaction > 0.05 for the majority of subgroups). A significant interaction was identified solely between race and EASIX (*P* < 0.001; *P* for interaction = 0.015), indicating that the impact of EASIX on 28-day all-cause mortality was more pronounced in White patients (Figure 4). Although this interaction was statistically significant (*P* ═ 0.015), potential confounding due to MNAR bias and mixed disease in the unidentified group precludes definitive conclusions regarding differences in pathological responses to EASIX between White and Non-White patients.

### Incremental value of EASIX compared to existing clinical scores

**Figure 5.**
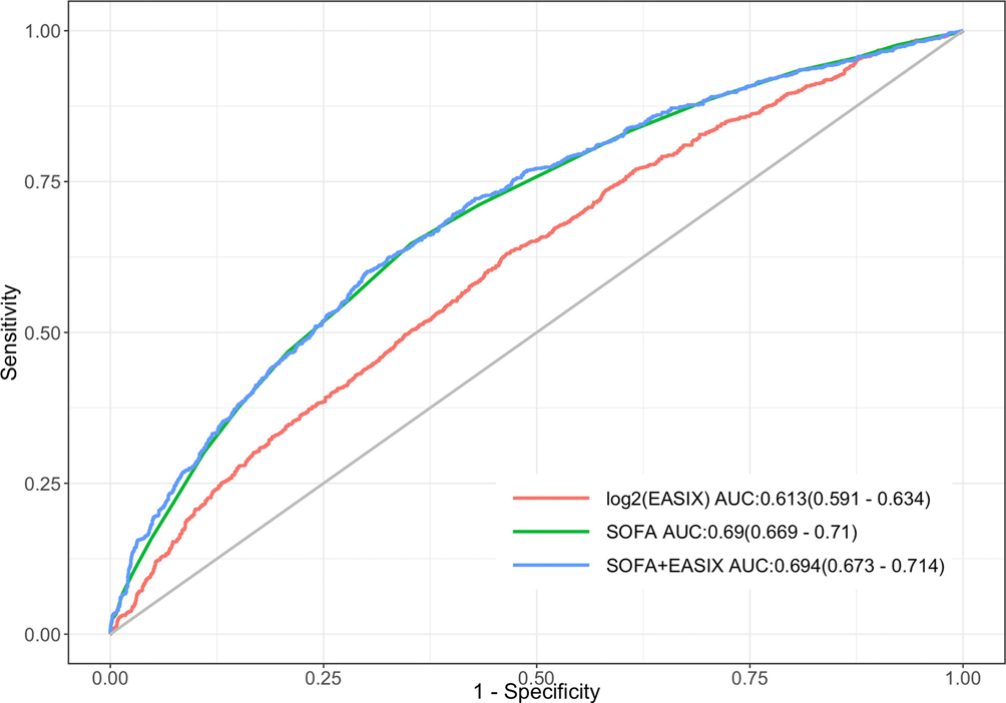
**ROC curves for prediction of 28-day all-cause mortality using log_2_(EASIX), SOFA, and the combined SOFA+EASIX model.** The AUC for log_2_(EASIX) was 0.613 (95% CI: 0.591–0.634), for SOFA 0.690 (95% CI: 0.669–0.710), and for SOFA+EASIX 0.694 (95% CI: 0.673–0.714). Abbreviations: ROC: Receiver operating characteristic; AUC: Area under the curve; CI: Confidence interval; EASIX: Endothelial activation and stress index; SOFA: Sequential Organ Failure Assessment.

**Figure 6.**
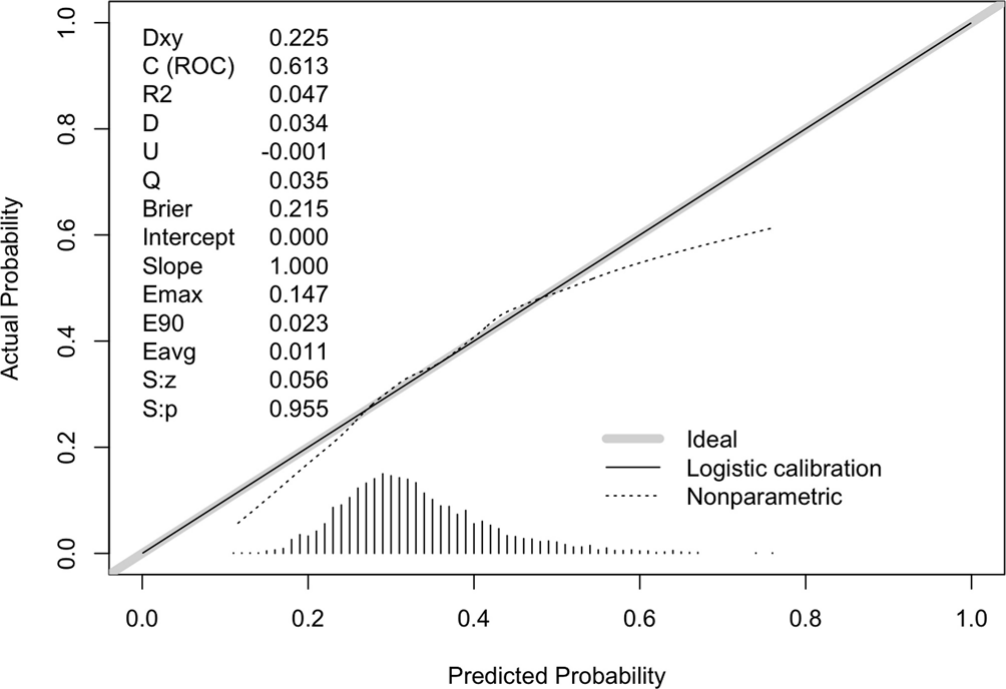
**Calibration plot of the SOFA+EASIX model for predicting 28-day all-cause mortality.** The gray line represents perfect agreement between predicted and observed risk, the solid black line shows logistic calibration, and the dotted line shows a nonparametric estimate. The calibration slope was 1.00 and the Brier score was 0.215, indicating good overall calibration. Abbreviations: EASIX: Endothelial activation and stress index; SOFA: Sequential Organ Failure Assessment; Brier: Brier score.

**Figure 7.**
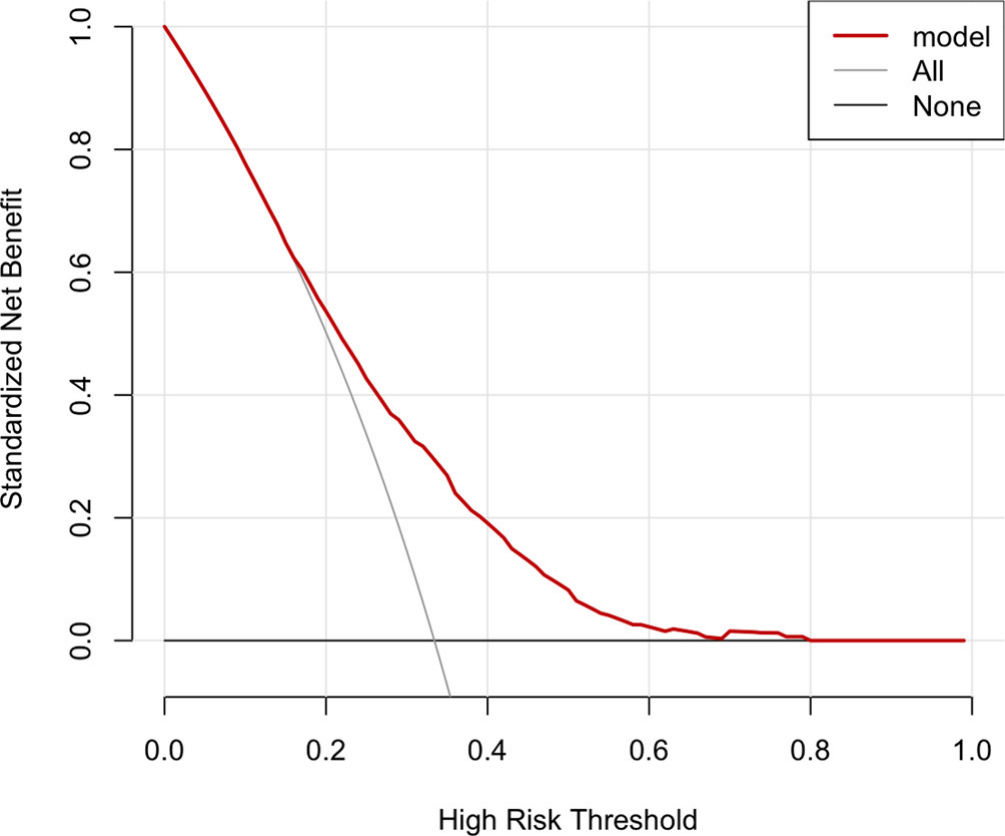
**Decision curve analysis of the EASIX-based prediction model for 28-day all-cause mortality.** The red line shows the standardized net benefit of the model across different high-risk thresholds, compared with strategies of treating all patients (light gray) or none (dark gray). The model yields greater net benefit than both reference strategies at lower threshold probabilities and approaches the “none” strategy at higher thresholds. Abbreviation: EASIX: Endothelial activation and stress index.

As depicted in Figure 5, the area under the curve (AUC) for log_2_(EASIX) was 0.613 (95% CI: 0.591–0.634), which is slightly lower than the AUC for the SOFA score (0.690, 95% CI: 0.669–0.710), indicating a lesser predictive ability relative to the SOFA score. However, the AUC for the combined model of SOFA and EASIX was 0.694 (95% CI: 0.673–0.714), suggesting that EASIX provides supplementary information to the SOFA score, enhancing its predictive performance. The DeLong test confirmed the statistical significance of these differences (EASIX + SOFA vs SOFA: *P* < 0.001). Additionally, the net reclassification improvement (NRI) and integrated discrimination improvement (IDI) were calculated (Table 3) to assess the incremental predictive value of the model incorporating EASIX with SOFA for 28-day mortality. Following the inclusion of EASIX, both NRI and IDI significantly increased (NRI (95% CI): 0.058 (0.030–0.103), *P* < 0.001; IDI (95% CI): 0.008 (0.003–0.015), *P* < 0.001). As illustrated in Figure 6, the calibration slope was 1.00 and the Brier score was 0.215, indicating good agreement between predicted probabilities and observed outcomes. However, the nonparametric curve slightly deviated from the ideal diagonal, suggesting discrepancies in predicted vs actual probabilities in low-to-medium risk populations, indicating potential overestimation or underestimation of mortality risk. The decision curve is presented in Figure 7. In the low threshold range (0.0–0.2), the model curve significantly surpassed the All and None curves, indicating that within this range, employing the EASIX model to identify and intervene in high-risk patients could yield substantial standardized net benefits. Conversely, in the medium-to-high threshold range (>0.4), the model curve gradually declined, approaching the None curve, suggesting that an excessively high threshold may reduce the model’s ability to identify high-risk patients, thereby diminishing net benefits.

**Table 3:** Incremental prognostic ability of EASIX for 28-day all-cause mortality

	**SOFA**	**SOFA+EASIX**	***P***
AUC (95% CI)	0.69 (0.669–0.71)	0.694 (0.673–0.714)	<0.001
NRI (95% CI)	Reference	0.058 (0.030–0.103)	<0.001
IDI (95% CI)	Reference	0.008 (0.003–0.015)	<0.001

Abbreviations: AUC: Area under the curve; CI: Confidence interval; NRI: Net reclassification improvement; IDI: Integrated discrimination improvement; SOFA: Sequential Organ Failure Assessment; EASIX: Endothelial activation and stress index.

### Sensitivity analysis

A sensitivity analysis based on complete cases was conducted using raw data without imputation or outlier handling to validate the robustness of the imputation and Winsorization methods. The core conclusions remained unaffected by outlier handling strategies (Supplementary Material 4). Sensitivity analysis was performed using the CRRT initiation time as the reference point, based on the first laboratory results obtained within 24 h after CRRT treatment. The results, presented in Supplementary Material 5, indicated that immortal-time bias had a minimal impact on the core effect size of this study. The association between EASIX and mortality in CRRT patients was robust and unaffected by the CRRT-anchored time-zero method. Given that 28-day mortality represents a short-term outcome, the odds ratio (OR) derived from logistic regression was highly comparable to the HR from the Cox model, further corroborating the conclusions from the perspective of cumulative risk. Additionally, logistic regression was performed (Supplementary Material 6), revealing core results that were highly consistent with the Cox analysis, thus validating the independent association between EASIX and 28-day mortality.

## Discussion

This study identified a significant relationship between EASIX and 28-day all-cause mortality in critically ill patients undergoing CRRT in the ICU. CRRT-treated patients with elevated EASIX values demonstrated a substantially increased risk of all-cause mortality within 28 days compared to those with lower values. In Models 1–3, each 1-unit increase in log_2_-EASIX was associated with a stable, slight upward trend in mortality risk. This association aligns with established characteristics of continuous biomarkers and prognostic outcomes. RCS results indicate that the risk effect of log_2_-EASIX can be accurately quantified using a single HR, making decomposition for result interpretation unnecessary and simplifying clinical applications. Furthermore, the model underwent validation via the Schoenfeld residual test, confirming the validity of the proportional hazards assumption for continuous variables. Sensitivity analyses also yielded consistent results. Incorporating log_2_-EASIX as a continuous variable in the SOFA-based model resulted in an increase in the AUC by 0.004 compared to the SOFA-only model. Both NRI and IDI significantly increased, indicating that continuous EASIX provides independent gradient risk information regarding mortality. The HR for each 1-unit increase in log_2_(EASIX) was compared between the minimally adjusted and fully adjusted models. Changes in HR were less than 5% and remained statistically significant, demonstrating that the predictive effect of EASIX on 28-day mortality was not significantly influenced by minor confounding factors. The minimally adjusted model accounted for the most critical confounding variables, and the slight decrease in HR in the fully adjusted model further validated the stability of the results, suggesting that the prognostic value of EASIX is robust in this study. RCS analysis revealed a linear positive association between log_2_(EASIX) and 28-day all-cause mortality. Additionally, subgroup analyses indicated that this association was generally consistent across most patient subgroups, with the exception of racial differences. The disparity in EASIX between White and Non-White groups is likely attributable to clinical factors, such as disease severity, rather than racial biological characteristics. Although these factors have been partially controlled in existing models, residual confounding may persist. Given potential deletion bias, the risk threshold of EASIX should not be adjusted for different racial groups in clinical practice. Instead, EASIX should be utilized as a general risk indicator and comprehensively assessed alongside the patient’s specific conditions (such as SOFA score and sepsis status).

EASIX serves as a straightforward and convenient predictive marker for multiple diseases, particularly in critical illness. In the context of cardiovascular critical illness, studies have shown that elevated EASIX scores correlate with microcirculatory failure and an increased likelihood of one-year all-cause mortality among individuals with cardiogenic shock [18]. Higher EASIX scores are also associated with increased 30-day mortality in individuals with acute myocardial infarction [16]. In addition, prior studies demonstrated that EASIX slightly outperformed existing scores in predicting 28-day mortality in individuals with liver failure (AUC = 0.771) and performed comparably to SOFA and acute physiology and chronic health evaluation II (APACHE-II) scores in assessing 3-month mortality (AUC = 0.791), while exhibiting considerable accuracy in predicting the need for hemodialysis (AUC = 0.833) [21]. The receiver operating characteristic (ROC) curves in this study suggest that for CRRT patients, the predictive capacity of EASIX is weaker than that of the SOFA score. However, the predictive ability of the combined model based on SOFA and EASIX is slightly enhanced compared to SOFA alone (AUC = 0.694 [95% CI: 0.673–0.714]), indicating that EASIX can provide supplementary information to SOFA. In severe acute pancreatitis, elevated EASIX values are significantly related to an increased risk of 28- and 90-day all-cause mortality, with ROC analysis showing that log_2_(EASIX) performs comparably to SOFA and systemic inflammatory response syndrome scores in prognostic prediction [14]. Similarly, elevated EASIX values are associated with a significantly higher risk of 28- and 90-day all-cause mortality among individuals with sepsis [15]. Furthermore, some studies suggest that distinct endothelial response patterns can differentiate sepsis patients at high risk of death [22]. In individuals with confirmed COVID-19, those with higher EASIX scores experienced significantly shorter overall survival compared to those with lower scores. EASIX also serves as a strong marker for ICU admission, AKI, in-hospital mortality, and the need for hemodialysis [13, 23]. Collectively, these findings underscore the expanding utility of EASIX. However, further validation in broader populations with endothelial dysfunction is warranted. To our knowledge, this study is the first to evaluate EASIX as a marker of endothelial dysfunction for predicting survival outcomes in individuals receiving CRRT.

The potential mechanisms underlying this association may involve several factors. Primarily, the population receiving CRRT consists of patients with severe AKI and critical sepsis, both closely linked to endothelial dysfunction. The renal vasculature comprises distinct endothelial cell populations, and damage caused by antibodies, immune cells, toxins, or inflammatory mediators can lead to acute or chronic kidney injury [24]. Sepsis and MODS frequently co-occur with AKI, and kidney injury is associated with disease progression and increased mortality [25, 26]. Our findings indicate that despite CRRT intervention, EASIX remains a significant prognostic indicator in individuals with sepsis and renal impairment. Additionally, EASIX reflects the severity of systemic inflammation. Elevated EASIX values are associated with increased serum levels of endothelial markers, including angiopoietin-2 (Ang-2), CXCL8, soluble thrombomodulin (sTM), and tumor necrosis factor-inducible gene 2. Additionally, higher EASIX values correlate with elevated inflammatory mediators, such as CXCL9, IL-18, and IL-18BPa [27]. In a study assessing EASIX in severely ill patients with advanced liver disease, it demonstrated superior diagnostic performance for detecting subclinical infections compared to conventional pro-inflammatory markers (AUC = 0.861, *P* < 0.001) [21]. Furthermore, low platelet counts included in EASIX indicate microcirculatory dysfunction. Most patients undergoing CRRT exhibit a hypercoagulable state prior to therapy, and the complex interactions among platelets, monocytes, neutrophils, coagulation pathways, and vascular endothelial cells are believed to promote microthrombus formation and retention within organ capillaries, resulting in ischemia and organ injury [28]. In patients receiving CRRT, the initiation of extracorporeal circulation during the early phase of treatment may induce transient hypotension and blood flow redistribution, further compromising microcirculation already at the threshold of hypoxia. Finally, LDH, as a marker of inflammation and oxidative stress, directly contributes to the promotion of apoptosis. Previous studies have confirmed that LDH is associated with in-hospital mortality in critically ill patients with AKI [29]. By integrating parameters reflecting inflammation, oxidative stress, and microthrombosis, EASIX may provide a more comprehensive prognostic assessment than LDH alone.

This study delineates the clinical context of CRRT, the pathological significance of endothelial dysfunction, the limitations of existing prognostic assessment systems, and the potential utility of EASIX. Evidence suggests that critically ill patients undergoing CRRT face a high risk of mortality, while traditional prognostic indicators inadequately capture endothelial dysfunction, a fundamental pathophysiological process. EASIX, as an emerging and accessible marker, has demonstrated robust prognostic value in sepsis and cardiovascular diseases. Its advantages in the prognostic evaluation of CRRT-treated patients can be summarized across three dimensions. Temporally, EASIX components can be measured daily, enabling real-time monitoring of endothelial status. Spatially, it integrates information on systemic endothelial injury, overcoming the limitations of single-organ function markers. From a pathophysiological perspective, EASIX directly reflects microcirculatory dysfunction, a hallmark of critical illness. Theoretically, early elevations of EASIX in CRRT-treated patients may indicate more severe systemic endothelial injury and a more challenging renal recovery, ultimately impacting 28-day survival.

However, some limitations of the present study must be acknowledged. First, due to its single-center design, selection bias cannot be entirely excluded. Second, certain critical information was unavailable in the database, including the timing of CRRT initiation and specific treatment parameters such as modality, dosage, anticoagulation strategy, and ultrafiltration settings, all of which could influence the study’s results. For instance, it remains unclear whether regional citrate anticoagulation affects endothelial function through its anti-inflammatory properties, thereby impacting EASIX. Similarly, whether repeated hemodynamic instability induced by high net ultrafiltration rates accelerates endothelial injury and results in rapid increases in EASIX remains to be determined. Furthermore, this study could not obtain endothelial-specific biomarker data. The mechanisms linking EASIX and the endothelium are discussed based solely on the indirect association of existing indicators and pathological references from external studies, lacking direct evidence from our cohort, which may lead to inference bias. Future research should incorporate endothelial biomarkers, such as Ang-2 and sTM in a multicenter prospective cohort to directly verify the association between EASIX and endothelial activation. Only prospective endothelial-specific data can more accurately define the pathological significance of EASIX. These questions should be addressed in multicenter prospective studies to further evaluate the feasibility and accuracy of EASIX.

## Conclusion

EASIX may serve as an independent predictor of short-term mortality risk among individuals receiving CRRT. Clinically, the findings of this study may inform the optimization of existing prognostic models and assist in the early identification of high-risk patients. Moreover, they provide a preliminary theoretical framework for the adjustment of individualized CRRT parameters and direct future efforts toward more accurate risk assessment and treatment strategy optimization in clinical practice, ultimately improving survival outcomes in this vulnerable population.

## Supplemental data

Supplemental data are available at the following link: https://www.bjbms.org/ojs/index.php/bjbms/article/view/13231/4074.

## Data Availability

The data in this study are derived from the Medical Information Mart for Intensive Care IV (MIMIC-IV) database (version 3.1). The datasets used and analysed during the current study are available from the corresponding author on reasonable request.
